# Ongoing pregnancy and healthy live births following very short ovarian stimulation of incidentally observed big antral follicles in oligoamenorrheic patients with extremely decreased ovarian reserve

**DOI:** 10.5935/1518-0557.20200095

**Published:** 2021

**Authors:** Volkan Turan, Meltem Sonmezer, Murat Sonmezer

**Affiliations:** 1 Health and Technology University School of Medicine, Istanbul, Turkey; 2 Private office, Ankara, Turkey; 3 Ankara University School of Medicine, Ankara, Turkey

**Keywords:** premature ovarian failure, random start ovarian stimulation, pregnancy rate

## Abstract

In the present case series our aim is to present seven patients with extremely decreased ovarian reserve and oligomenorrhea, conceived with *in vitro* fertilization following a very short ovarian stimulation of incidentally detected big antral follicles. The study included women pursuing *in vitro* fertilization due to premature ovarian failure risk. When an incidental growing antral follicle was detected under ultrasound, immediate ovarian stimulation was initiated if the blood estradiol, luteinizing hormone and progesterone levels were correlated. Serum anti-Mullerian hormone measurements of all patients were consistent with extremely diminished ovarian reserve (ranged between 0.01 and 0.09ng/ml) and FSH levels varied between 13-104IU/l. The mean stimulation length ranged between 2-4 days. A total of 8 oocytes were retrieved; 6 MII, 1 GV and 1 degenerated. All 6 MII oocytes were fertilized with intracytoplasmic sperm injection. Two patients conceived after fresh embryo transfer, whereas the one conceived following frozen thawed embryo transfer. The ongoing pregnancy rate was 50% per transfer, and two of them resulted in a healthy live birth. In conclusion, close monitoring of oligoamenorrheic infertile patients who are at high risk of imminent ovarian failure using ultrasound and blood hormone levels is very important. Albeit low, the possibility of having a healthy pregnancy following “a very short ovarian stimulation” in such a specific patient group is emphasized.

## INTRODUCTION

Premature ovarian failure (POF) is a loss of normal ovary function before age 40 (Kovanci & Schutt, 2015). It affects approximately: one in 10,000 women by age 20; one in 1,000 women by age 30; one in 100 women by age 40 (Coulam *et al*., 1986). A woman with POF may still have follicles, but very few. Women with POF can still have a menstruation, but most of the time the periods are irregular. If POF occurs in women who are planning to conceive, infertility can be a problem (Rebar, 2009).

Recent evidence indicates that there are multiple follicle recruitment waves during a normal menstrual cycle; hence, there usually is more than one time window in a single menstrual cycle to start ovarian stimulation (Oktay *et al*., 2008). Therefore, random start ovarian stimulation protocols have been developed. These protocols were previously used in patients with cancer, who have time constraints to start chemotherapy, and in patients who were poor responders to ovarian stimulation (Sonmezer *et al*., 2011; Zhang *et al*., 2018; Xu & Li, 2013). Since menstrual irregularity is common in women with POF, random start ovarian stimulation can be initiated during regular monitoring if a developing follicle is detected under ultrasound.

We previously described ongoing pregnancies from early retrieval of prematurely developing antral follicles in two patients with severely diminished ovarian reserve, who were refractory to aggressive ovarian stimulation (Sonmezer *et al*., 2009). Here we present seven cases with extremely decreased ovarian reserve and oligomenorrhea, to report pregnancy outcomes after *in vitro* fertilization following a very short ovarian stimulation of incidentally detected big antral follicles with a diameter between 14-17 mm.

## Case Presentations

### Case 1

A 31-year-old woman presented to our clinic seeking fertility treatment. Her anti-Mullerian hormone (AMH) and follicle stimulating hormone (FSH) levels were 0.09 ng/ml and 42 IU/l, respectively. She had had one previous IVF failure, and 2 cancelled cycles due to unsuccessful ovarian stimulation. Her last period was 41 days ago and an incidental developing antral follicle was detected with a diameter of 16.5 mm on ultrasound examination. Her blood estradiol (E2) was 97pg/ml, luteinizing hormone (LH) was 2.7 IU/l, and progesterone was 1.4 pg/ml. After 2 days of stimulation with highly purified human menotropin (hp-hMG) 150 IU/d (Menopur, Ferring) along with gonadotropin releasing hormone (GnRh) antagonist (Cetrotide, Merck Serono) to prevent premature ovulation, the follicle was triggered with recombinant human chorionic gonadotropin (Ovitrelle, 250 µg, Merk Serono). One oocyte was retrieved and fertilized. A high quality embryo was frozen on day 5. Two months later, following endometrial preparation blastocyst transfer resulted in pregnancy and live birth.

### Case 2

The second patient was operated for breast cancer 7 years ago, and had been on tamoxifen treatment for 5 years. When she applied to our clinic for infertility, she was 37 years old and she was not under tamoxifen treatment for a year. Although she did not take chemotherapy, her AMH and FSH levels were 0.03 ng/ml and 13 IU/l, respectively and she had irregular menstrual cycles. Her previous menstruation was 2.5 months ago. During her initial work-up, a 17 mm antral follicle was detected on the 4^th^ day of her menstrual cycle. Based on blood estrogen level consistent with the developing follicle (145 pg/ml), ovarian stimulation was initiated with hpHMG 150 IU/day, together with GnRH antagonist treatment, and maintained for 2 days. Serum progesterone on the day of hCG trigger was 1.3 ng/ml. After ovulation trigger with recombinant hCG on day 6 (Ovitrelle, 250 µg, Merk Serono), one oocyte was retrieved and fertilized. A fresh blastocyst transfer was performed on day 13^th^ of the menstrual cycle, resulting in an ongoing healthy pregnancy.

### Case 3

A 31-year-old woman with an AMH level <0.01 ng/ml was admitted to our clinic with a diagnosis of infertility and premature ovarian failure. Her serum FSH levels ranged between 49-104 IU/l. She had menstrual irregularities for a year; therefore, she was put on cyclic estradiol valerate and norgestrel treatment. In addition, she had been using dehydroepiandrosterone (DHEA) for four months (75 mg/d). During the regular follow up, a dominant follicle with 17 mm diameter was incidentally detected on the 4^th^ day of the cycle and ovarian stimulation was performed with 150 IU of hp-hMG in conjunction with GnRH antagonist. Her FSH, LH, estradiol and progesterone levels were 11 IU/l, 1.8 IU/l, 308 pg/ml and 0.5 ng/ml, respectively. After 2 days of ovarian stimulation, ovulation trigger was performed using 250 µg of recombinant hCG (Ovitrelle, Merk Serono), and one oocyte was retrieved. After successful fertilization with ICSI, one embryo developed to blastocyst, and fresh transfer on day 13^th^ day of the menstrual cycle resulted in a healthy pregnancy and live birth.

### Case 4

A 30-year-old woman with irregular menstrual cycles ranging between 2-3 months presented with infertility. Her FSH level was 24.7 IU/l. Her last menstrual period was 67 days ago. On first examination, we found a 15 mm developing follicle. Her serum E2 level was 112 pg/ml and progesterone was 1.7 ng/ml. After 4 days of ovarian stimulation, together with daily 10 mg of medroxyprogesterone acetate (Tarlusal, Deva, Turkey), one degenerated oocyte was retrieved following hCG trigger.

### Case 5

The patient was 36 years old and she had had menstrual irregularity for 2 years. Her FSH levels ranged between 17.4 and 23.1 IU/l. She was on levothyroxine treatment for Hashimoto thyroiditis. She had one previous IVF attempt with no response to ovarian stimulation. During initial workup in our clinic, a 16mm ovarian follicle was detected on her 21^th^ day of the menstrual cycle. Her serum estradiol level was 189 pg/ml, LH was 3.2 IU/l, and progesterone was 1.2 ng/ml. After two days of ovarian stimulation with hp-hMG (150 IU/day), one MII oocyte was retrieved. A high quality day-3 frozen/thawed embryo was transferred ending up with a biochemical pregnancy.

### Case 6

The patient was 36 years old and she had menstrual irregularity for a year. Her AMH level was 0.01 ng/ml. On the second day of menstruation, a 14 mm ovarian follicle was detected under transvaginal ultrasound. Her blood estradiol level was 116 pg/ml, LH was 5.3 IU/l and progesterone was 1.0 ng/ml. After four days of ovarian stimulation with hMG (300 IU/day), one MII oocyte was retrieved following hCG trigger. Her MII oocyte was fertilized and a high quality day-5 embryo was obtained and frozen after preimplantation genetic screening performed using next generation sequencing. The embryo was found euploid, and the embryo transfer resulted in an ectopic pregnancy.

### Case 7

A 43-year-old woman with low ovarian reserve presented a follicle with a mean diameter of 17.5 mm on her day 2 of the cycle. Her serum E2, LH and AMH levels were 318 pg/ml and 11 IU/l, 0.09 ng/ml respectively. After two days of ovarian stimulation with hMG (150 IU/day), one MII oocyte was retrieved following hCG trigger. A day-3 high-grade embryo was frozen due to inappropriate endometrial lining for transfer.

Collectively, the mean stimulation length and total gonadotropin dose were 2-4 days and 300-1200 IU, respectively. Out of 8 retrieved oocytes, 6 MII oocytes were fertilized and 6 embryos were obtained. The ongoing pregnancy rate per transfer was 50% and 2 of them resulted in a healthy delivery [Table 1].

**Table 1:** Demographics, cycle and pregnancy outcomes of all patients with poor ovarian reserve

**Patients**	**Age (years)**	**AMH level (ng/ml)**	**Mean follicle diameter at COH start**	**COH start day**	**Serum estradiol levels at start (pg/dl)**	**Serum FSH levels at start (IU/ml)**	**Total stimulation length (days)**	**Number of embryos obtained**	**Serum estradiol levels on trigger day (pg/dl)**	**Transfer type**	**Pregnancy**
**Case 1**	31	0.09	16.5	41 days after last period	97	42	2	1 (Day 5)	217	Frozen	Live birth
**Case 2**	37	0.03	17	2.5 months after last period	145	13	2	1 (Day 5)	407	Fresh	Ongoing pregnancy
**Case 3**	31	<0.01	17	On cycle day 4		49	2	1 (Day 5)	-	Fresh	Live birth
**Case 4**	30	0.04	15	67 days after last period	112	24.7	4	-	358	-	-
**Case 5**	36	0.03	16	21th day of the menstrual cycle	189	17.4	2	1 (Day 3)	313	Frozen	Biochemical pregnancy
**Case 6**	36	0.01	14	On cycle day 2	116	-	4	1 (Day 5)	213	Frozen	Ectopic pregnancy
**Case 7**	43	0.09	17.5	On cycle day 2	318	-	2	1 (Day 3)	-	Frozen	Not transferred yet

## DISCUSSION

In this study, we presented *in vitro* fertilization outcomes of seven women with a diagnosis of imminent ovarian failure who had menstrual irregularities due to diminished ovarian reserve. Finally, out of six patients that underwent embryo transfer, four conceived: two resulted in healthy live births, one in ongoing pregnancy and one in ectopic pregnancy. In the last patient, one top quality day-3 embryo was frozen due to inappropriate endometrium.

For the presented cases, it is worth mentioning that all but one were diagnosed with premature ovarian failure. A woman with premature ovarian failure may still have follicles; however, due to diminished ovarian reserve they may experience irregular menstrual periods. Although traditional concept of folliculogenesis supports the recruitment of an antral follicle cohort in the late luteal phase of the preceding menstrual cycle as a result of rising FSH levels, recent evidence indicates that there are multiple follicle recruitment waves during a menstrual cycle (Oktay et al., 2008); therefore, close monitoring of ovaries using ultrasound has aroused increased interest, especially in women with diminished ovarian reserve. It was reported that synchronization of follicular waves with random start ovarian stimulation results in normal fertilization rates and embryo quality (Bianchi *et al*., 2010). Moreover, patients with decreased ovarian reserve are likely to have big antral follicles due to advanced follicular maturation in the very early days of follicular phase. These follicles should not be misdiagnosed as persistent luteal cysts. In the present study, all patients were followed up twice a month for 6 months to observe follicular growth. Since they were oligoamenorrheic, GnRH antagonists were started straightforwardly in conjunction with a minimal dose of gonadotropins to prevent premature LH surge, when a developing follicle was detected incidentally under ultrasound.

It can be questioned whether the oocytes and embryos obtained and frozen with late follicular or luteal phase-start ovarian stimulation will result in comparable pregnancy rates with those originating from conventional stimulation cycles. Many studies have been performed to investigate cycle and pregnancy outcomes after in vitro fertilization. The existing data, and as confirmed by the findings of the current cases, demonstrated favorable pregnancy rates after late follicular or luteal start of ovarian stimulation (Kuang *et al*., 2014). Furthermore, no increased risk was indicated for live-birth defects in random start stimulation cycles compared with conventional stimulation cycles (Chen *et al*., 2015).

The synchronized development of a viable embryo and a receptive endometrium is crucial for successful implantation to take place. Random start ovarian stimulation may pose to asynchrony between endometrium and embryo; hence, it is possible that fresh ET will result in implantation failure. Therefore, we evaluated serum progesterone levels of patients on trigger day to decide whether to perform fresh or frozen/thawed embryo transfer. If serum progesterone level is lower than 1.5 ng/ml on trigger day together with a clear trilaminar endometrial sign, fresh embryo transfer was performed (Ashmita *et al*., 2017).

The role of preimplantation genetic screening (PGS) in women with decreased ovarian reserve is controversial. In women with normal ovarian reserve, it was demonstrated that PGS with the use of comprehensive chromosome screening technology increases pregnancy rates by improving embryo selection, and it prevents multiple pregnancies (Chen *et al*., 2015). However, particularly in patients with POF, there are no supernumerary embryos in contrast to normal/high responders, and whether PGS provides any benefit can be challenged. Furthermore, it should be kept in mind that there is a risk of damage to the embryo during the removal of cells. Since it was hypothesized that quality and quantity of the follicular pool are directly related (Baird *et al*., 2005). La Marca *et al*. (2017) investigated whether ovarian reserve, measured through serum anti-Mullerian hormone may determine the rate and number of euploid blastocysts in IVF cycles. They found that there is a strong positive age-independent relationship between AMH level and the rate of euploid blastocysts. In contrast, in a more recent study, AMH level was found to be associated with increased risk of embryo aneuploidy only in women ≥35 years of age (Jiang *et al*., 2018). Both studies had a retrospective design and had relatively small sample sizes. Moreover, a recent study demonstrated that low oocyte yield in IVF treatment was not associated with a higher risk of trisomic pregnancy (Honorato *et al*., 2017). Therefore, RCTs with larger sample sizes are required for PGS in women with POF.

In conclusion, meticulous follow up of oligoamenorrheic patients with extremely decreased ovarian reserve seeking fertility is of crucial importance. The emergence of big antral follicles in the very early days of the menstrual cycle, or those found incidentally on a random day, should not be misdiagnosed as a physiological ovarian cyst, and these patients should not be refrained from undergoing ART. After a very short ovarian stimulation, IVF can be utilized and fresh or frozen-thawed embryo transfer can be performed following a careful assessment of blood hormone results and endometrial pattern. Even though a 50% high pregnancy rate may be a coincidental finding, the possibility of a healthy pregnancy is worth mentioning in such extreme clinical situations with a close follow up.
